# Accelerating Wright–Fisher Forward Simulations on the Graphics Processing Unit

**DOI:** 10.1534/g3.117.300103

**Published:** 2017-08-02

**Authors:** David S. Lawrie

**Affiliations:** Los Angeles, California 90034

**Keywords:** GPU, Wright–Fisher model, simulation, population genetics

## Abstract

Forward Wright–Fisher simulations are powerful in their ability to model complex demography and selection scenarios, but suffer from slow execution on the Central Processor Unit (CPU), thus limiting their usefulness. However, the single-locus Wright–Fisher forward algorithm is exceedingly parallelizable, with many steps that are so-called “embarrassingly parallel,” consisting of a vast number of individual computations that are all independent of each other and thus capable of being performed concurrently. The rise of modern Graphics Processing Units (GPUs) and programming languages designed to leverage the inherent parallel nature of these processors have allowed researchers to dramatically speed up many programs that have such high arithmetic intensity and intrinsic concurrency. The presented GPU Optimized Wright–Fisher simulation, or “GO Fish” for short, can be used to simulate arbitrary selection and demographic scenarios while running over 250-fold faster than its serial counterpart on the CPU. Even modest GPU hardware can achieve an impressive speedup of over two orders of magnitude. With simulations so accelerated, one can not only do quick parametric bootstrapping of previously estimated parameters, but also use simulated results to calculate the likelihoods and summary statistics of demographic and selection models against real polymorphism data, all without restricting the demographic and selection scenarios that can be modeled or requiring approximations to the single-locus forward algorithm for efficiency. Further, as many of the parallel programming techniques used in this simulation can be applied to other computationally intensive algorithms important in population genetics, GO Fish serves as an exciting template for future research into accelerating computation in evolution. GO Fish is part of the Parallel PopGen Package available at: http://dl42.github.io/ParallelPopGen/.

The GPU is commonplace in today’s consumer and workstation computers and provides the main computational throughput of the modern supercomputer. A GPU differs from a computer’s CPU in a number of key respects, but the most important differentiating factor is the number and type of computational units. While a CPU for a typical consumer laptop or desktop will contain anywhere from two to four very fast, complex cores, GPU cores are in contrast relatively slow and simple. However, there are typically hundreds to thousands of these slow and simple cores in a single GPU. Thus, CPUs are low latency processors that excel at the serial execution of complex, branching algorithms. Conversely, the GPU architecture is designed to provide high computational bandwidth, capable of executing many arithmetic operations in parallel.

The historical driver for the development of GPUs was increasingly realistic computer graphics for computer games. However, researchers quickly latched on to their usefulness as tools for scientific computation, particularly for problems that were simply too time consuming on the CPU due to sheer number of operations that had to be computed but where many of those operations could in principle be computed simultaneously. Eventually programming languages were developed to exploit GPUs as massive parallel processors and, over time, the GPU hardware has likewise evolved to be more capable for both graphics and computational applications.

Population genetics analysis of single nucleotide polymorphisms (SNPs) is exceptionally amenable to acceleration on the GPU. Beyond the study of evolution itself, such analysis is a critical component of research in medical and conservation genetics, providing insight into the selective and mutational forces shaping the genome as well as the demographic history of a population. One of the most common analysis methods is the site frequency spectrum (SFS), a histogram where each bin is a count of how many mutations are at a given frequency in the population.

SFS analysis is based on the precepts of the Wright–Fisher process (Fisher 1930; Wright 1938), which describes the probabilistic trajectory of a mutation’s frequency in a population under a chosen evolutionary scenario. The defining characteristic of the Wright–Fisher process is forward time, nonoverlapping, discrete generations with random genetic drift modeled as a binomial distribution dependent on the population size and the frequency of a mutation (Fisher 1930; Wright 1938). On top of this foundation can be added models for selection, migration between populations, mate choice and inbreeding, and linkage between different loci, etc. For simple scenarios, an approximate analytical expression for the expected proportion of mutations at a given frequency in the population, the expected SFS, can be derived (Fisher 1930; Wright 1938; Kimura 1964; Sawyer and Hartl 1992; Williamson *et al.* 2004). This expectation can then be compared to the observed SFS of real data, allowing for parameter fitting and model testing (Williamson *et al.* 2004; Machado *et al.* 2017). However, more complex scenarios do not have tractable analytical solutions, approximate or otherwise. One approach is to simulate the Wright–Fisher process forward in time to build the expected frequency distribution or other population genetic summary statistics (Hernandez 2008; Messer 2013; Thornton 2014; Ortega-Del Vecchyo *et al.* 2016). Because of the flexibility inherent in its construction, the Wright–Fisher forward simulation can be used to model any arbitrarily complex demographic and selection scenario (Hernandez 2008; Carvajal-Rodriguez 2010; Hoban *et al.* 2012; Messer 2013; Thornton 2014; Ortega-Del Vecchyo *et al.* 2016). Unfortunately, because of the computational cost, the use of such simulations to analyze polymorphism data are often prohibitively expensive in practice (Carvajal-Rodriguez 2010; Hoban *et al.* 2012). The coalescent looking backward in time and approximations to the forward single-locus Wright–Fisher algorithm using diffusion equations provide alternative, computationally efficient methods of modeling polymorphism data (Hudson 2002; Gutenkunst *et al.* 2009). However, these effectively limit the selection and demographic models that can be ascertained and approximate the Wright–Fisher forward process (Gutenkunst *et al.* 2009; Carvajal-Rodriguez 2010; Ewing and Hermisson 2010; Hoban *et al.* 2012). Thus, by speeding up forward simulations, we can use more complex and realistic demographic and selection models to analyze within-species polymorphism data.

Single-locus Wright–Fisher simulations based on the Poisson Random Field model (Sawyer and Hartl 1992) ignore linkage between sites and simulate large numbers of individual mutation frequency trajectories forward in time to construct the expected SFS. Exploiting the naturally parallelizable nature of the single-locus Wright–Fisher algorithm, these forward simulations can be greatly accelerated on the GPU. Written in the programming language CUDA (Nickolls *et al.* 2008), a C/C++ derivative for NVIDIA GPUs, GO Fish allows for accurate, flexible simulations of SFS at speeds orders of magnitude faster than comparative serial programs on the CPU. As a programming library, GO Fish can be run in standalone scripts or integrated into other programs to accelerate single-locus Wright–Fisher simulations used by those tools.

## Methods

### Algorithm

In a single-locus Wright–Fisher simulation, a population of individuals can be represented by the set of mutations segregating in that population, specifically by the frequencies of the mutant, derived alleles in the population. Under the Poisson Random Field model, these mutations are completely independent of each other and new mutational events only occur at nonsegregating sites in the genome (*i.e.*, no multiple hits) (Sawyer and Hartl 1992).

**Figure 1:**
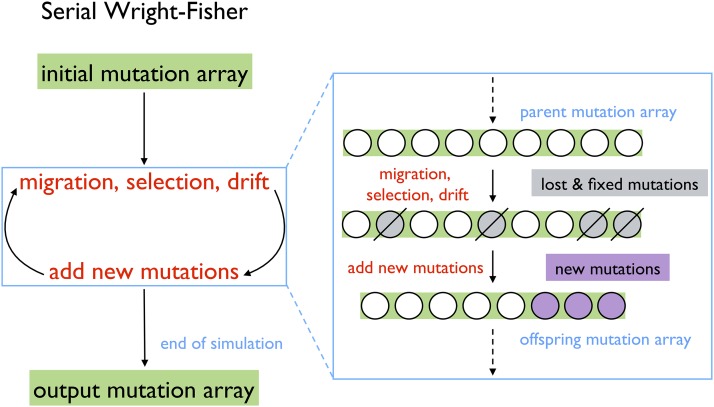
Serial Wright–Fisher algorithm. Mutations are the “unit” of simulation for the single-locus Wright–Fisher algorithm. Thus, a generation of organisms is represented by an array of mutations and their frequency in the (each) population (if there are multiple in the simulation). There are several options for how to initialize the mutation array to start a simulation: a blank mutation array, the output of a previous simulation run, or mutation–selection equilibrium (for details, see File S1). Simulating each discrete generation first consists of calculating the new allele frequency of each mutation, one at a time, where those mutations that become lost or fixed are discarded. Next, new mutations are added to the array, again, one at a time. The resulting offspring array of mutation frequencies becomes the parent array of the next generation and the cycle is repeated until the end of the simulation when the final mutation array is output.

Figure 1 sketches the algorithm for a typical, serial Wright–Fisher simulation, starting with the initialization of an array of mutation frequencies. From one discrete generation time step to the next, the change in any given mutation’s frequency is dependent on the strength of selection on that mutation, migration from other populations, the percent of inbreeding, and genetic drift. Unlike the others listed, inbreeding is not directly a force for allele frequency change, but rather it modifies the effectiveness of selection and drift. Frequencies of 0 (lost) and 1 (fixed) are absorbing boundaries such that if a mutation becomes fixed or lost across all extant populations, it is removed from the next generation’s mutation array. New mutations arising stochastically throughout the genome are then added to the mutation array of the offspring generation, replacing those mutations lost and fixed by selection and drift. As the offspring become the parents of the next generation, the cycle repeats until the final generation of the simulation.

**Figure 2:**
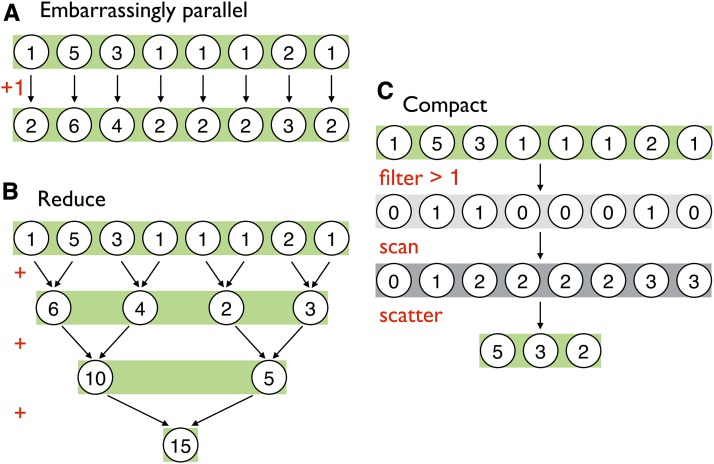
Common parallel algorithms. Illustrative examples of three classes of common parallel algorithms implemented using simple operations and an eight-element, integer array. (A) Embarrassingly parallel algorithms are those that can be computed independently and thus simultaneously on the GPU. The given example, adding one to every element of an array, can be done concurrently to all array elements. In GO Fish, calculating new mutation frequencies and adding new mutations to population are both embarrassingly parallel operations. (B) Reduce is a fundamental parallel algorithm in which all the elements of an array are reduced to a single value using a binary operator, such as in the above summation over the example array (Harris 2007a). This algorithm takes advantage of the fact that in each time step half of the sums can be done independently while synchronized communication is necessary to combine the results of previous time steps. In total, log_2_(8) = 3 time steps are required to reduce the example array. (C) Compact is a multi-step algorithm that allows one to filter arrays on the GPU (Billeter *et al.* 2009). In an embarrassingly parallel step, the algorithm presented above first creates a new Boolean array of those elements that passed the filter predicate (*e.g.*, *x* > 1). Then a “scan” is performed on the Boolean array. Scan is similar in concept to reduce, wherein for each time step half of the binary operations are independent, but it is a more complex parallel algorithm that creates a running sum over the array rather than condensing the array to a single value [see Harris *et al.* (2007b)]. This running sum determines the new index of each element in the original array being filtered and the size of the new array. Those elements that passed the filter are then scattered to their new indices of the now smaller, filtered array. Compact is used in GO Fish to filter out fixed and lost mutations. GPU, Graphics Processing Unit.

While the details of how a GPU organizes computational work are quite intricate (Nickolls *et al.* 2008), the vastly oversimplified version is that a serial set of operations is called a thread and the GPU can execute many such threads in parallel. With completely unlinked sites, every simulated mutation frequency trajectory is independent of every other mutation frequency trajectory in the simulation. Therefore, the single-locus Wright–Fisher algorithm is trivially parallelized by simply assigning a thread to each mutation in the mutation array; when simulating each discrete generation, both calculating the new frequency of alleles in the next generation and adding new mutations to next generation are embarrassingly parallel operations (Figure 2A). This is the parallel ideal because no communication across threads is required to make these calculations. A serial algorithm has to calculate the new frequency of each mutation one by one, and the problem is multiplied where there are multiple populations as these new frequencies have to be calculated for each population. For example, in a simulation with 100,000 mutations in a given generation and three populations, 300,000 sequential passes through the functions governing migration, selection, and drift are required. However, in the parallel version, this huge number of iterations can theoretically be compressed to a single step in which all the new frequencies for all mutations are computed simultaneously. Similarly, if there are 5000 new mutations in a generation, a serial algorithm has to add each of those 5000 new mutations one at a time to the simulation. The parallel algorithm can, in theory, add them all at once. Of course, a GPU only has a finite number of computational resources to apply to a problem and thus this ideal of executing all processes in a single time step is never truly realizable for a problem of any substantial size. Even so, parallelizing migration, selection, drift, and mutation on the GPU results in dramatic speedups relative to performing those same operations serially on the CPU. This is the main source of GO Fish’s improvement over serial, CPU-based Wright–Fisher simulations.

One challenge to the parallelization of the Wright–Fisher algorithm is the treatment of mutations that become fixed or lost. When a mutation reaches a frequency of 0 (in all populations, if multiple) or 1 (in all populations, if multiple), that mutation is forever lost or fixed. Such mutations are no longer of interest to maintain in memory or process from one generation to the next. Without removing lost and fixed mutations from the simulation, the number of mutations being stored and processed would simply continue to grow as new mutations are added each generation. When processing mutations one at a time in the serial algorithm, removing mutations that become lost or fixed is as trivial as simply not adding them to the next generation and shortening the mutation array in the next generation by one each time. This becomes more difficult when processing mutations in parallel. As stated before: the different threads for different mutations do not communicate with each other when calculating the new mutation frequencies simultaneously. Therefore, any given mutation/thread has no knowledge of how many other mutations have become lost or fixed that generation. This in turn means that when attempting to remove lost and fixed mutations while processing mutations in parallel, there is no way to determine the size of the next generation’s mutation array or where in the offspring array each mutation should be placed.

**Figure 3:**
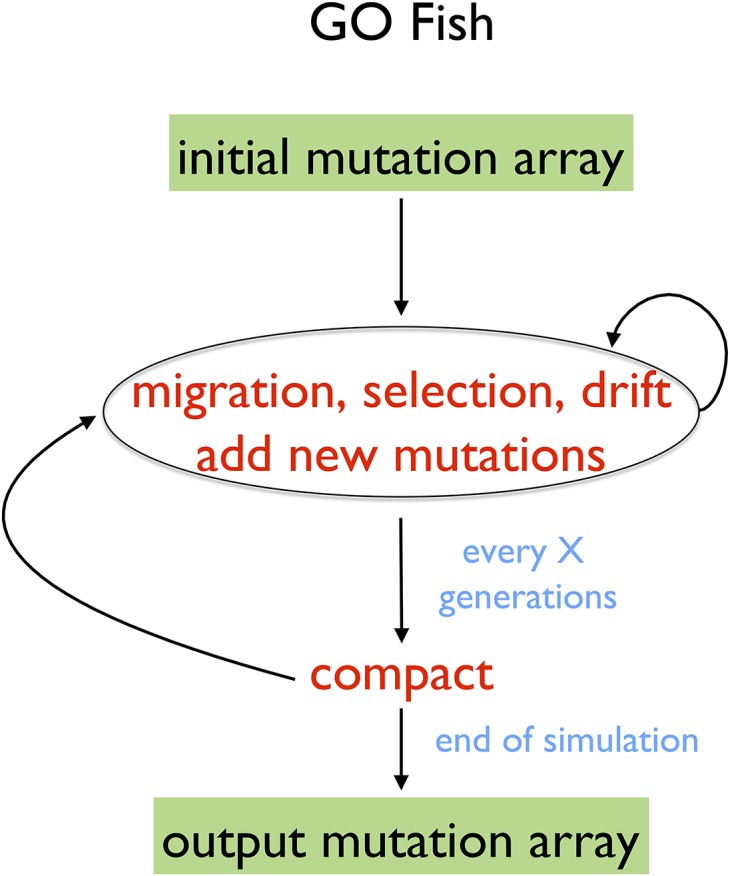
GO Fish algorithm. Both altering the allele frequencies of mutations from parent to child generation and adding new mutations to the child generation are embarrassingly parallel operations (see Figure 2A) that are greatly accelerated on the GPU. Further, as independent operations, adding new mutations and altering allele frequencies can be done concurrently on the GPU. In comparison to serial Wright–Fisher simulations (Figure 1), GO Fish includes an extra compact step (see Figure 2C) to remove fixed and lost mutations every X generations. Until compaction, the size of the mutation array grows by the number of new mutations added each generation. Before the simulation ends, the program compacts the mutation array one final time. GPU, Graphics Processing Unit.

One solution to the above problems is the algorithm “compact” (Billeter *et al.* 2009), which can filter out lost and fixed mutations while still taking advantage of the parallel nature of GPUs (Figure 2C). However, compaction is not embarrassingly parallel, as communication between the different threads for different mutations is required, and it involves a lot of moving elements around in GPU memory rather than intensive computation. Thus, it is a less efficient use of the GPU as compared to calculating allele frequencies. As such, a nuance in optimizing GO Fish is how frequently to remove lost and fixed mutations from the active simulation. Despite the fact that computation on such mutations is wasted, calculating new allele frequencies is so fast that not filtering out lost and fixed mutations every generation and temporarily leaving them in the simulation actually results in faster runtimes. Eventually of course, the sheer number of lost and fixed mutations overwhelms even the GPU’s computational bandwidth and they must be removed. How often to compact for optimal simulation speed can be ascertained heuristically and is dependent on the number of mutations each generation in the simulation and the attributes of the GPU the simulation is running on. Figure 3 illustrates the algorithm for GO Fish, which combines parallel implementations of migration, selection, drift, and mutation with a compacting step run every X generations and again before the end of the simulation.

### The population genetics model of GO Fish

A more detailed description of the implementation of the Wright–Fisher algorithm underlying GO Fish, with derivations of the equations below, can be found in Supplemental Material, File S1. Table 1 provides a glossary of the variables used in the simulation.

**Table 1:** Glossary of simulation terms

Variable	Definition
μ(*j*,*t*)	Mutation rate per site per chromosome for population *j* at time *t*
s(*j*,*t*,*x*)*^a^*	Selection coefficient for a mutation at frequency *x* in population *j* at time *t*
h(*j,t*)*^b^*	Dominance of allele for population *j* at time *t*
F(*j*,*t*)*^c^**^,^**^d^*	Inbreeding coefficient in population *j* at time *t*
N(*j*,*t*)*^d^*	Number of individuals in population *j* at time *t*
N_e_(*j*,*t*)*^d^*	Effective number of chromosomes in population *j* at time *t*
m(*k*,*j*,*t*)	Migration: proportion of chromosomes from population *k* in population *j* at time *t*
*L*	Number of sites in simulation

a *s* = 0 (neutral), 0 > *s* > −1 (purifying selection), and 0 < *s* (positive selection).

b *h* = 1 (dominant), *h* = 0 (recessive), *h* > 1 / *h* < 0 (over-/underdominant), and 0 < *h* < 1 (codominant).

c *F* = 1 (haploid), *F* = 0 (diploid), and 0 < *F* < 1 (inbred diploid).

d *N_e_* = 2 × *N* / (1 + *F*).

The simulation can start with an empty initial mutation array, with the output of a previous simulation run, or with the frequencies of the initial mutation array in mutation–selection equilibrium. Starting a simulation as a blank canvas provides the most flexibility in the starting evolutionary scenario. However, to reach an equilibrium start point requires a “burn-in,” which may be quite a large number of generations (Ortega-Del Vecchyo *et al.* 2016). To save time, if a starting scenario is shared across multiple simulations, then the postburn-in mutation array can be simulated beforehand, stored, and input as the initial mutation array for the next set of simulations. Alternatively, the simulation can be initialized in a calculable, approximate mutation–selection equilibrium state, allowing the simulation of the evolutionary scenario of interest to begin essentially immediately. λ_μ_(*x*) is the expected (mean) number of mutations at a given frequency, *x*, in the population at mutation–selection equilibrium and can be calculate via the following equation:

μ=μ(j,0), s(x)=s(j,0,x), etc...

(1) $$\lambda_{\mu }(x)=\frac{2\mu L}{x(1-x)e^{-N_{e}\text{​}s(x)x\left\{\left(2h+(1-2h)x\right)(1-F)+2F\right\}}}\times \frac{\int_{x}^{1}e^{-N_{e}\text{​}s(y)y\left\{\left(2h+(1-2h)y\right)(1-F)+2F\right\}}dy}{\int_{0}^{1}e^{-N_{e}\text{​}s(y)y\left\{\left(2h+(1-2h)y\right)(1-F)+2F\right\}}dy}$$

The derivation for Equation 1 can be found in File S1 (Equation 1–6 in File S1). The numerical integration required to calculate λ_μ_(*x*) has been parallelized and accelerated on the GPU. To start the simulation, the actual number of mutations at each frequency is determined by draws from the Inverse Poisson distribution with mean and variance λ_μ_(*x*). This numerical initialization routine can handle most of the equilibrium evolutionary scenarios the main simulation is capable of itself, a major exception being those cases with migration between multiple populations. Given the number of cases covered by the above integration technique, this is likely to be the primary method to start a GO Fish simulation in a state of mutation–selection equilibrium.

After initialization begins the cycle of adding new mutations to the population and calculating new frequencies for currently segregating mutations. The number of new mutations introduced in each population *j*, for each generation *t* is Poisson distributed with mean *N_e_*μ*L* in accordance with the assumptions of the Poisson Random Field Model. These new mutations start at frequency 1/*N_e_* in the simulation. Meanwhile, the SNP frequencies of the extant mutations in the current generation *t* and population *j* are modified by the forces of migration (I.), selection (II.), and drift (III.) to produce the new frequencies of those mutations in generation *t + 1*.

$$x_{t,j}\overset{\text{I.}}{\to }x_{mig}\overset{\text{II.}}{\to }x_{mig,sel}\overset{\text{III.}}{\to }x_{mig,sel,drift}=x_{t+1,j}$$

I. GO Fish uses a conservative model of migration (Nagylaki 1980) where the new allele frequency, *x_mig_*, in population *j* is the average of the allele frequency in all the populations weighted by the migration rate from each population to population *j*. II. Selection further modifies the expected frequency of the mutations in population *j* according to Equation 2 below:

s(x)=s(j,t,x), h=h(j,t), etc...

(2) $$x_{mig,sel}=\frac{x_{mig}^{2}s(x_{mig})+x_{mig}(1-x_{mig})s(x_{mig})(F+h-hF)+x_{mig}}{x_{mig}^{2}s(x_{mig})+x_{mig}(1-x_{mig})s(x_{mig})(F+2h-2hF)+1}$$

The derivation for Equation 2 can be found in File S1 (Equation 8–13 in File S1). The variable *x_mig,sel_* represents the expected frequency of an allele in generation *t + 1*. III. Drift, which is modeled as a binomial random deviation with mean *N_e_x_mig,sel_* and variance *N_e_x_mig,sel_*(1-*x_mig,sel_*), then acts on top of the deterministic forces of migration and selection to produce the ultimate frequency of the allele in the next generation, *t + 1*, in population *j*, *x_t+1,j_*. Then the cycle repeats.

### Data availability

**Figure 4:**
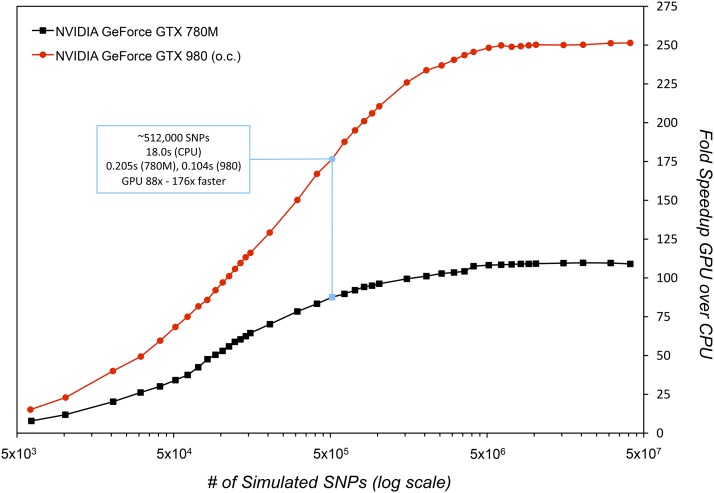
Performance gains on GPU relative to CPU. The above figure plots the relative performance of GO Fish, written in CUDA, to a basic, serial Wright–Fisher simulation written in C++. The two programs were run both on a 2013 iMac with an NVIDIA GeForce GTX 780M mobile GPU, 1536 at 823 MHz cores, (black line) and an Intel Core i7 4771 CPU at 3.9 GHz and a self-built Linux-box with a factory-overclocked NVIDIA GeForce GTX 980 GPU, 2048 at 1380 MHz cores, and an Intel Core i5 4690K CPU at 3.9 GHz (red line). Full compiler optimizations (−O3 –fast-math) were applied to both serial and parallel programs. Each dot represents a simulation run plotted by the number of SNPs in its final generation. The serial program was run on the ∼10,000, ∼100,000, and ∼1 × 10^6^ SNP scenarios. As the speed of the CPU-based program is linear on the number of simulated SNPs, the resulting runtimes of 0.4, 3.9, and 38.7 sec were then linearly rescaled to estimate runtimes for serial simulations with differing numbers of final SNPs. The two Intel processors have identical speeds on single-threaded, serial tasks, which also allows for direct comparison between the two GPU results. Consumer GPUs like the 780M and 980 need to warm up from idle and load the CUDA context. So, to obtain accurate runtimes on the GPU, GO Fish timings were done after 10 runs had finished and then the average of another 10 runs was taken for each data point. The GO Fish compacting rate was hand-optimized for each number of simulated SNPs, for each processor (Table S1). CPU, Central Processor Unit; GPU, Graphics Processing Unit; SNP, single nucleotide polymorphism.

**Figure 5:**
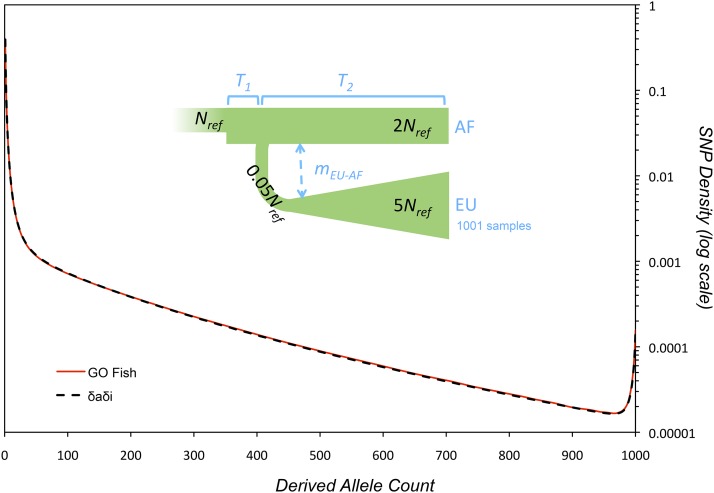
Validation of GO Fish simulation results against δaδi. A complex demographic scenario was chosen as a test case to compare the GO Fish simulation against an already established site frequency spectrum (SFS) method, δaδi (Gutenkunst *et al.* 2009). The demographic model is from a δaδi code example for the Yoruba-Northern European (AF-EU) populations. Using δaδi parameterization to describe the model, the ancestral population, in mutation–selection equilibrium, undergoes an immediate expansion from *N_ref_* to 2*N_ref_* individuals. After time *T_1_* (= 0.005) the population splits into two with a constant, equivalent migration, *m_EU-AF_* (= 1) between the now split populations. The second (EU) population undergoes a severe bottleneck of 0.05*N_ref_* when splitting from the first (AF) population, followed by exponential growth over time *T_2_* (= 0.045) to size 5*N_ref_*. The SFS (black dashed line) above is of weakly deleterious, codominant mutations (2*N_ref_s* = −2, *h* = 0.5) where 1001 samples were taken of the EU population. The spectrum was then normalized by the number of segregating sites. The corresponding GO Fish parameters for the evolutionary scenario, given a mutation rate of 1 × 10^−9^ per site, 2 × 10^9^ sites, and an initial population size, *N_ref_*, of 10,000, are: *T_1_* = 0.005 × 2*N_ref_* = 100 generations, *T_2_* = 900 generations, *m_EU-AF_* = 1/(2*N_ref_*) = 0.00005, 2*N_ref_s* = −4, *h* = 0.5, and *F* = 0. As in δaδi, the population size/time can be scaled together and the simulation will generate the same normalized spectra (Gutenkunst *et al.* 2009). Using the aforementioned parameters, a GO Fish simulation ends with ∼3 × 10^6^ mutations, of which ∼560,000 are sampled in the SFS. The red line reporting GO Fish results is the average of 50 such simulations; the dispersion of those 50 simulations is reported in Figure S1. Each simulation run on the NVIDIA GeForce GTX 980 took roughly the same time to generate the SFS as δaδi did [grid size = (110,120,130), time-step = 10^−3^] on the Intel Core i7 4771, just < 0.7 sec.

The library of parallel APIs, the Parallel PopGen Package, of which GO Fish is a member, is hosted on GitHub at https://github.com/DL42/ParallelPopGen. In the Git repository, the code generating Figure 4, Figure 5, and Figure S1 is in the folders examples/example_speed (Figure 4) and examples/example_dadi (Figure 5 and Figure S1) along with example outputs. The companion serial Wright–Fisher simulation, serial_SFS_code.cpp, is provided in examples/examples_speed, as is a help file, serial_SFS_code_help.txt, and makefile, makefile_serial. Table S1, referenced in Figure 4, can also be found under the folder documentation/ in the GitHub repository uploaded as excel file 780M_980_Intel_speed_results.xlsx. The API manual is at http://dl42.github.io/ParallelPopGen/.

## Results and Discussion

To test the speed improvements from parallelizing the Wright–Fisher algorithm, GO Fish was compared to a serial Wright–Fisher simulation written in C++. Each program was run on two computers: an iMac and a self-built Linux-box with equivalent Intel Haswell CPUs, but very different NVIDIA GPUs. Constrained by the thermal and space requirements of laptops and all-in-one machines, the iMac’s NVIDIA 780M GPU (1536 cores at 823 MHz) is slower and older than the NVIDIA 980 (2048 cores at 1380 MHz) in the Linux-box. For a given number of simulated populations and number of generations, a key driver of execution time is the number of mutations in the simulation. Thus, many different evolutionary scenarios will have similar runtimes if they result in similar numbers of mutations being simulated each generation. As such, to benchmark the acceleration provided by parallelization and GPUs, the programs were run using a basic evolutionary scenario while varying the number of expected mutations in the simulation. The utilized scenario is a simple, neutral simulation, starting in mutation–selection equilibrium, of a single, haploid population with a constant population size of 200,000 individuals over 1000 generations and a mutation rate of 1 × 10^−9^ mutations per generation per individual per site. With these other parameters held constant, varying the number of sites in the simulation adjusts the number of expected mutations for each of the benchmark simulations.

As shown in Figure 4, accelerating the Wright–Fisher simulation on a GPU results in massive performance gains on both an older, mobile GPU like the NVIDIA 780M and a newer, desktop-class NVIDIA 980 GPU. For example, when simulating the frequency trajectories of ∼500,000 mutations over 1000 generations, GO Fish takes ∼0.2 sec to run on a 780M as compared to ∼18 sec for its serial counterpart running on the Intel i5/i7 CPU (@3.9 GHz), a speedup of 88-fold. On a full, modern desktop GPU like the 980, GO Fish runs this scenario ∼176× faster than the strictly serial simulation and only takes ∼0.1 sec to run. As the number of mutations in the simulation grows, more work is tasked to the GPU and the relative speedup of GPU to CPU increases logarithmically. Eventually though, the sheer number of simulated SNPs saturates even the computational throughput of the GPUs, producing linear increases in runtime for increasing SNP counts, like for serial code. Thus, eventually, there is a flattening of the fold performance gains. This plateau occurs earlier for 780M than for the more powerful 980 with its more and faster cores. Executed serially on the CPU, a huge simulation of ∼4 × 10^7^ SNPs takes roughly 24 min to run *vs.* only ∼13/5.7 sec for GO Fish on the 780M/980, an acceleration of > 109/250-fold. While not benchmarked here, the parallel Wright–Fisher algorithm is also trivial to partition over multi-GPU setups in order to further accelerate simulations.

Tools employing the single-locus Wright–Fisher framework are widely used in population genetics analyses to estimate selection coefficients and infer demography [see Gutenkunst *et al.* (2009), Garud *et al.* (2015), Ortega-Del Vecchyo *et al.* (2016), Jackson *et al.* (2017), Kim *et al.* (2017), Koch and Novembre (2017), Machado *et al.* (2017) for examples]. Often, these tools employ either a numerically solved diffusion approximation, or even the simple analytical function, to generate the expected SFS of a given evolutionary scenario, which can then be used to calculate the likelihood of producing an observed SFS. The model parameters of the evolutionary scenario are then fitted to the data by maximizing the composite likelihood. With GO Fish, forward simulation can generate the expected spectra. To validate these expected spectra, the results of GO Fish simulations were compared against δaδi (Gutenkunst *et al.* 2009) for a complex evolutionary scenario involving a single population splitting into two, exponential growth, selection, and migration (Figure 5). The spectra generated by each program are identical. Interestingly, the two programs also had essentially identical runtimes for this scenario and hardware (Figure 5). In general, the relative compute time will vary depending on the simulation size and population scaling for GO Fish, the grid size and time-step for δaδi (Gutenkunst *et al.* 2009), and the simulation scenario and computational hardware for both.

For maximum-likelihood and Bayesian statistics as for parametric bootstraps and C.I.s, hundreds, thousands, and even tens of thousands of distinct parameter values may need to be simulated to yield the needed statistics for a given model. Multiplying this by the need to often consider multiple evolutionary models as well as nonparametric bootstrapping of the data, a single serial simulation run on a CPU taking only 18 sec, as in the simple simulation of ∼500,000 SNPs presented in Figure 4, can add up to hours, even days of compute time. Moreover, and in contrast to the approximating analytical or numerical solutions typically employed, simulating the expected SFS introduces random noise around the “true” SFS of the scenario being modeled. Figure S1 demonstrates how increasing the number of simulated SNPs increases the precision of the simulation and therefore of the ensuing likelihood calculations. Simulating tens of millions of SNPs, wherein a single run on the CPU can take nearly half-an-hour, can be imperative to obtain a high-precision SFS needed for certain situations. Thus, the speed boost from parallelization on the GPU in calculating the underlying, expected SFS greatly enhances the practical utility of simulation for many current data analysis approaches. The speed and validation results demonstrate that, now with GO Fish, one can not only track allele trajectories in record time, but also generate SFS by using forward simulations in roughly the same time-frame as by solving diffusion equations. Just as importantly, GO Fish achieves the increase in performance without sacrificing flexibility in the evolutionary scenarios that it is capable of simulating.

GO Fish can simulate mutations across multiple populations for comparative population genomics, with no limits to the number of populations allowed. Population size, migration rates, inbreeding, dominance, and mutation rate are all user-specifiable functions capable of varying over time and between different populations. Selection is likewise a user-specifiable function parameterized not only by generation and population, but also by allele frequency, allowing for the modeling of frequency-dependent selection as well as time-dependent and population-specific selection. By tuning the inbreeding and dominance parameters, GO Fish can simulate the full range of single-locus dynamics for both haploids and diploids with everything from outbred to inbred populations and overdominant to underdominant alleles. GPU-accelerated Wright–Fisher simulations thus provide extensive flexibility to model unique and complex demographic and selection scenarios beyond what many current site frequency spectrum analysis methods can employ.

Paired with a coalescent simulator, GO Fish can also accelerate the forward simulation component in forward-backward approaches [see Ewing and Hermisson (2010) and Nakagome *et al.* (2016)]. In addition, GO Fish is able to track the age of mutations in the simulation, providing an estimate of the distribution of the allele ages, or even the age by frequency distribution, for mutations in an observed SFS. Further, the age of mutations is one element of a unique identifier for each mutation in the simulation, which allows the frequency trajectory of individual mutations to be tracked through time. This ability to sample ancestral states and then track the mutations throughout the simulation can be used to contrast the population frequencies of polymorphisms from ancient DNA with those present in modern populations for powerful population genetics analyses (Bank *et al.* 2014). By accelerating the single-locus forward simulation on the GPU, GO Fish broadens the capabilities of SFS analysis approaches in population genetic studies.

Across the field of population genetics and evolution, there exist a wide range of computationally intensive problems that could benefit from parallelization. The algorithms presented and discussed in Figure 2 represent a subset of the essential parallel algorithms, which more complex algorithms modify or build upon. Applications of these parallel algorithms are already wide-ranging in bioinformatics: motif finding (Ganesan *et al.* 2010), global and local DNA and protein alignment (Vouzis and Sahinidis 2011; Liu *et al.* 2011, 2013; Zhao and Chu 2014), short read alignment and SNP calling (Klus *et al.* 2012; Luo *et al.* 2013), haplotyping and the imputation of genotypes (Chen *et al.* 2012), analysis for genome-wide association studies (Chen 2012; Song *et al.* 2013), and mapping phenotype to genotype and epistatic interactions across the genome (Chen and Guo 2013; Cebamanos *et al.* 2014). In molecular evolution, the basic algorithms underlying the building of phylogenetic trees and analyzing sequence divergence between species have likewise been GPU-accelerated (Suchard and Rambaut 2009; Kubatko *et al.* 2016). Further, there are parallel methods for general statistical and computational methods, like Markov Chain Monte Carlo and Bayesian analysis, useful in computational evolution and population genetics (Suchard *et al.* 2010; Zhou *et al.* 2015). GO Fish is itself part of the larger Parallel PopGen Package, a planned compendium of tools for accelerating the calculation of many different population genetics statistics on the GPU, including SFS and likelihoods. This larger package is currently under development; the results in Figure 5 make use of a prototype library, Spectrum, to generate SFS statistics from GO Fish simulations on the GPU.

Future work on the single-locus Wright–Fisher algorithm will include extending the parallel structure of GO Fish to allow for multiple alleles as well as multiple mutational events at a site, relaxing one of the key assumptions of the Poisson Random Field (Sawyer and Hartl 1992). At present, neither running simulations with long divergence times between populations nor any scenario where the number of extant mutations in the simulation rises to too high a proportion of the total number of sites is theoretically consistent with the Poisson Random Field model underpinning the current version of GO Fish. Beyond GO Fish, solving Wright–Fisher diffusion equations in programs like δaδi (Gutenkunst *et al.* 2009) can likewise be sped up through parallelization on the GPU (Lions *et al.* 2001; Komatitsch *et al.* 2009; Micikevicius 2009; Tutkun and Edis 2012).

Unfortunately, while the effects of linkage and linked selection across the genome can be mitigated in analyses using a single-locus framework (Gutenkunst *et al.* 2009; Coffman *et al.* 2016; Machado *et al.* 2017), these effects cannot be examined and measured while assuming independence among sites. Expanding from the study of independent loci to modeling the evolution of haplotypes and chromosomes, simulations with the coalescent framework or forward Wright–Fisher algorithm with linkage can also be accelerated on GPUs. The coalescent approach has already been shown to benefit from parallelization over multiple CPU cores [see Montemuiño *et al.* (2014)]. While Montemuiño *et al.* (2014) achieved their speed boost by running multiple independent simulations concurrently, they noted that parallelizing the coalescent algorithm itself may also accelerate individual simulations over GPUs (Montemuiño *et al.* 2014). Likewise, multiple independent runs of the full forward simulation with linkage can be run concurrently over multiple cores and the individual runs might themselves be accelerated by parallelization of the forward algorithm. The forward simulation with linkage has many embarrassingly parallel steps, as well as those that can be refactored into one of the core parallel algorithms. The closely related genetic algorithm, used to solve difficult optimization problems, has already been parallelized and, under many conditions, greatly accelerated on GPUs (Pospichal *et al.* 2010; Hofmann *et al.* 2013; Limmer and Fey 2016). However, not all algorithms will benefit from parallelization and execution on GPUs; the real-world performance of any parallelized algorithm will depend on the details of the implementation (Hofmann *et al.* 2013; Limmer and Fey 2016). While the extent of the performance increase will vary from application to application, each of these represent key algorithms whose potential acceleration could provide huge benefits for the field (Carvajal-Rodriguez 2010; Hoban *et al.* 2012).

These potential benefits extend to lowering the cost barrier for students and researchers to run intensive computational analyses in population genetics. The GO Fish results demonstrate how powerful even an older, mobile GPU can be at executing parallel workloads, which means that GO Fish can be run on everything from GPUs in high-end compute clusters to a GPU in a personal laptop and still achieve a great speedup over traditional serial programs. A batch of single-locus Wright–Fisher simulations that might have taken a 100 CPU-hr or more to complete on a cluster can be done, with GO Fish, in 1 hr on a laptop. Moreover, graphics cards and massively parallel processors in general are evolving quickly. While this paper has focused on NVIDIA GPUs and CUDA, the capability to take advantage of the massive parallelization inherent in the Wright–Fisher algorithm is the key to accelerating the simulation and in the high-performance computing market there are several avenues to achieve the performance gains presented here. For instance, OpenCL is another popular low-level language for parallel programming and can be used to program NVIDIA, AMD, Altera, Xilinx, and Intel solutions for massively parallel computation, which include GPUs, CPUs, and even Field Programmable Gate Arrays (Stone *et al.* 2010; Czajkowski *et al.* 2012; Jha *et al.* 2015). The parallel algorithm of GO Fish can be applied to all of these tools. Whichever platform(s) or language(s) researchers choose to utilize, the future of computation in population genetics is massively parallel and exceedingly fast.

## Supplementary Material

Supplemental material is available online at www.g3journal.org/lookup/suppl/doi:10.1534/g3.117.300103/-/DC1.
