# Supplementary material for: Accelerating Wright–Fisher Forward Simulations on the Graphics Processing Unit
Source: G3 (Bethesda). 2017 Aug 2;7(9):3229–36. doi: 10.1534/g3.117.300103 (PMC5592947; doi:10.1534/g3.117.300103)
Supplement: Supplementary file 1 [file 3229FileS1.pdf]

Appendix – Parallel Wright-Fisher Simulation Details

Simulation Initialization

Simulations can be initialized in one of three ways: 1) a blank canvas, 2) from the results of a previous simulation, and 3) mutation-selection equilibrium. Starting a simulation as a blank canvas provides the most flexibility in what evolutionary state the simulation begins and thus any evolutionary scenario can be simulated from the beginning. However, as the simulation starts with no mutations present, a “burn-in” time is necessary to reach the point where the simulation of the scenario of interest can begin. The number of “burn-in” generations may be quite long, particularly to reach any kind of equilibrium state where selection, mutation, migration, and drift are all in balance and the number of mutations being fixed and lost is equal to the number of new mutations in the population(s). To save time, if a starting scenario is shared across multiple simulations, then the post-burn-in mutation array can be simulated beforehand, stored, and input as the initial mutation array for the next set of simulations.

Another way to jump start the simulation is by assuming all extant populations are in mutation-selection balance at the beginning of the simulation. Under general mutation-selection equilibrium (MSE), the proportion of mutations at every frequency in the population can be calculated via numerical integration over a continuous frequency diffusion approximation (see Kimura 1964). While this constrains the starting evolutionary state to mutation-selection equilibrium, this allows one to then start simulating the selection and demographic scenario of interest immediately. Due to current limitations of the MSE model in GO Fish, the mutation-selection equilibrium scenario does not, as of yet, include migration from other populations or random fluctuations in selection intensity – nor can the code calculate the number of generations ago a mutation at frequency *x* is expected to have arisen at. Instead all mutations in the initial mutation array said to have arisen at time t = 0. The model is detailed below:

Using the glossary from Table 1, for any given population j at time t = 0:

$$\mu = \mu(j,0), \quad \mathrm{s}(x) = \mathrm{s}(j,0,x), \quad \text{etc...}$$

From Kimura p. 220-222 (Kimura 1964):

$$1)\ \lambda_\mu(x) = \frac{2\mu L}{N_e \mathrm{V}(x)\mathrm{G}(x)} \frac{\int_x^1 \mathrm{G}(y)dy}{\int_0^1 \mathrm{G}(y)dy}$$

$$2)\ \mathrm{G}(x) = e^{-\int \frac{2\mathrm{M}(x)}{\mathrm{V}(x)}dx}$$

$$3)\ \mathrm{V}(x) = x(1-x)/N_e$$

$$\text{where } N_e = 2N/(1+F)$$

$\lambda_\mu(x)$ is the expected (mean) number of mutations at a given frequency, *x*, in the population at mutation-selection equilibrium. V(*x*) and M(*x*) are the contribution of drift and selection respectively to the rate of change of a mutation’s frequency at frequency *y* in the population. Since this is an allele-based simulation, I use the equilibrium value of the effective number of chromosomes, N_e, to account for inbreeding amongst *N* individuals.

$$4a)\ \mathrm{M}(x) = \mathrm{M}_{dip}(x)(1-F) + \mathrm{M}_{hap}(x)F$$
$$4b)\ \mathrm{M}_{hap}(x) = \mathrm{s}(x)x(1-x)$$
$$4c)\ \mathrm{M}_{dip}(x) = \mathrm{s}(x)\left\{h + (1-2h)x\right\}x(1-x)$$

The total rate of frequency change is the average of the rate of change of the effective haploid proportion of the population and the effective diploid proportion of the population weighted by *F*.

$$5)\ \mathrm{G}(x) = e^{-\int \frac{2\mathrm{M}(x)}{\mathrm{V}(x)}dx} = e^{-\int \frac{2\mathrm{M}_{dip}(x)(1-F)+2\mathrm{M}_{hap}(x)F}{\mathrm{V}(x)}dx} = e^{-N_e \mathrm{s}(x)x\left\{(2h+(1-2h)x)(1-F)+2F\right\}}$$

Substituting eq. 3 and 5 into eq. 1 yields:

$$6)\ \lambda_\mu(x) = \frac{2\mu L}{x(1-x)e^{-N_e \mathrm{s}(x)x\left\{(2h+(1-2h)x)(1-F)+2F\right\}}} \frac{\int_x^1 e^{-N_e \mathrm{s}(y)y\left\{(2h+(1-2h)y)(1-F)+2F\right\}}dy}{\int_0^1 e^{-N_e \mathrm{s}(y)y\left\{(2h+(1-2h)y)(1-F)+2F\right\}}dy}$$

More familiar versions of eq. 6 can be derived by assuming neutrality or by assuming no frequency-dependent selection and either codominance or haploid/completely inbred individuals.

$$\text{if } \mathrm{s}(x) = 0 \ \ \forall x \in (0,1) \text{ (neutral)} \rightarrow \ \lambda_\mu(x) = 2\mu L/x$$
$$\text{if } \mathrm{s}(x) = s \ \forall x \in (0,1) \text{ and } (h = 0.5 \text{ or } F = 1) \ \rightarrow \lambda_\mu(x) = \frac{2\mu L}{x(1-x)} \frac{1-e^{-2N_e s(1-x)}}{1-e^{-2N_e \mathrm{s}}}$$
$$\text{where if } h = 0.5 \text{ (codominant)} \rightarrow \ N_e = 2N/(1+F)$$
$$\text{where if } F = 1 \text{ (haploid)} \rightarrow \ N_e = N$$

I approximate the integrals in eq. 6 using trapezoidal numerical integration and use the *scan* parallel algorithm implemented in CUB 1.6.4 (Merrill 2016) to accelerate the integration on the GPU*. $\lambda_\mu(x)$ is the expected (mean) number of mutations. To determine the actual number of mutations at a given frequency, *x*, I generate random numbers from the Inverse Poisson distribution with mean $\lambda_\mu(x)$ using the following procedure:

I. Random number generator Philox (Salmon *et al.* 2011) generates a uniform random number between 0 and 1.
II. If $\lambda_\mu(x) \leq 6$, then that uniform variable is fed into the exact Inverse Poisson CDF.
III. If $\lambda_\mu(x) > 6$, then a Normal approximation to the Poisson is used.

Adding all the new mutations at every frequency to the starting mutation array is an embarrassingly parallel problem. Thus, combined with the parallel numerical integration for the definite integral components of eq. 6, initializing the simulation at mutation-selection equilibrium is overall greatly accelerated on the GPU relative to serial algorithms on the CPU.

***An Aside About Numerical Precision, GPUs, and Numerical Integration:** For a bit of background, CPUs employ a Floating-point Processor Unit with 80-bits of precision for serial floating-point computation, which then quickly translates the result into double-precision (64-bit) for the CPU registers. Thus, CPU programs, including the serial Wright-Fisher simulation, are often written with double-precision performance in mind. In contrast, most consumer GPU applications are geared towards single-precision (32-bit) computation (e.g. graphics) and many consumer GPUs have relatively poor double-precision performance. More expensive, professional-grade workstation GPUs often have far better double-precision performance than their consumer counterparts. As the Wright-Fisher simulation does not actually require 64-bits of precision for its calculations, GO Fish has been written with 32-bits of precision computation in mind. This is even true of the MSE Integration step where the naturally pair-wise summation of parallel scanning mitigates the round-off error when performing large numbers of consecutive sums in 32-bit (Higham 1993). That said, the mutation frequencies stored in the simulation have only single-precision floating-point accuracy. Experiments using CPU serial Wright-Fisher simulations showed consistent results between storing mutation frequencies with 32-bits vs. 64-bits of precision.

Steps to Calculate New Allele Frequencies

Migration, selection, and drift determine the frequency of an allele in the next generation, x_{t+1}, based on its current frequency, x_t. Migration and selection are deterministic forces whereas drift introduces binomial random chance. While these three steps can, in principle, be done in any order, their order in the simulation is as follows:

I. Migration
II. Selection (with Inbreeding)
III. Drift (with Inbreeding)

$$x_{t,j} \xrightarrow{\text{I.}} x_{mig} \xrightarrow{\text{II.}} x_{mig,sel} \xrightarrow{\text{III.}} x_{mig,sel,drift} = x_{t+1,j}$$

I. Migration

Using the glossary from Table 1, in population *j* at time *t*:

$$\mathrm{m}(k) = \mathrm{m}(k,j,t),$$
$$x_{t,k} \equiv \text{ freq. of allele in pop. } k \text{ at time } t,$$
$$x_{mig} = x_{mig,j} \equiv \text{ freq. of allele in pop. } j \text{ after migration},$$

$$7)\ x_{mig} = \sum_k \mathrm{m}(k) x_{t,k}$$
$$\text{where } \mathrm{m}(j) = 1 - \sum_{k \neq j} \mathrm{m}(k)$$

GO Fish uses a conservative model of migration (Nagylaki 1980). The new allele frequency in population *j* is the average of the allele frequency in all the populations weighted by the migration rate from each population, to population *j*. And the migration rate is specified by the proportion of chromosomes from population *k* in population *j*.

II. Selection (with Inbreeding)

In population *j* at time *t*:

$x_{mig} = x_{mig,j} \equiv$ freq. of allele after migration, $y_{mig} = 1 - x_{mig}$,

$x_{mig,sel} = x_{mig,sel,j} \equiv$ freq. of allele after migration and selection,

$P_{AA}, P_{Aa}, P_{aa} \equiv$ frequency of genotype AA, Aa, and aa,

$\mathrm{s}(x) = \mathrm{s}(j,t,x)$, $\mathrm{h} = \mathrm{h}(j,t)$,

$w = w_j \equiv$ average pop. j fitness, $n = n_j \equiv$ average pop. j fitness of allele A

selection model		
P_{AA}<br>1+s(x)	P_{Aa}<br>1+hs(x)	P_{aa}<br>1

$$8)\ n = P_{AA}\left(1+\mathrm{s}(x_{mig})\right) + P_{Aa}\left(1+h\,\mathrm{s}(x_{mig})\right)/2$$

$$9)\ w = P_{AA}\left(1+\mathrm{s}(x_{mig})\right) + P_{Aa}\left(1+h\,\mathrm{s}(x_{mig})\right) + P_{aa}$$

$$10)\ x_{mig,sel} = n/w$$

Like with M(x) in eq. 4, w and n are a weighted average of the effective haploid (inbred) and diploid (outbred) portions of the chromosome population. Diploid genotype frequencies assume random mating and Hardy-Weinberg equilibrium (Hardy 1908, Weinberg 1908).

$$11a)\ w = w_{dip}(1-F) + w_{hap}F$$

$$11b)\ w_{dip} = x_{mig}^2\left(1+\mathrm{s}(x_{mig})\right) + 2x_{mig}y_{mig}\left(1+h\,\mathrm{s}(x_{mig})\right) + y_{mig}^2$$

$$11c)\ w_{hap} = x_{mig}\left(1+\mathrm{s}(x_{mig})\right) + y_{mig}$$

$$11d)\ w = x_{mig}^2\,\mathrm{s}(x_{mig}) + x_{mig}(1-x_{mig})\mathrm{s}(x_{mig})(F+2h-2hF) + 1$$

Following the same logic as above:

$$12a)\ n = n_{dip}(1-F) + n_{hap}F$$

$$12b)\ n = x_{mig}^2\,\mathrm{s}(x_{mig}) + x_{mig}(1-x_{mig})\mathrm{s}(x_{mig})(F+h-hF) + x_{mig}$$

Substituting eq. 11*d* and 12*b* into eq. 10 yields:

$$13)\ x_{mig,sel} = \frac{x_{mig}^2\,\mathrm{s}(x_{mig}) + x_{mig}(1-x_{mig})\mathrm{s}(x_{mig})(F+h-hF) + x_{mig}}{x_{mig}^2\,\mathrm{s}(x_{mig}) + x_{mig}(1-x_{mig})\mathrm{s}(x_{mig})(F+2h-2hF) + 1}$$

Again, like for eq. 6, more familiar forms of eq. 13 may be derived under certain assumptions such as neutrality, haploid/inbred individuals, and completely outbred diploids.

$$\text{if } s(x_{mig}) = 0 \; \forall x \in (0,1) \text{ (neutral)} \rightarrow x_{mig,sel} = x_{mig}$$

$$\text{if } F = 1 \text{ (haploid)} \rightarrow x_{mig,sel} = \frac{x_{mig}\, s(x_{mig}) + x_{mig}}{x_{mig}\, s(x_{mig}) + 1}$$

$$\text{if } F = 0 \text{ (diploid)} \rightarrow x_{mig,sel} = \frac{x_{mig}^2\, s(x_{mig})(1-h) + x_{mig}(h\, s(x_{mig}) + 1)}{x_{mig}^2\, s(x_{mig})(1-2h) + 2x_{mig} h\, s(x_{mig}) + 1}$$

III. Drift (with Inbreeding)

For population *j* in generation *t*:

$$x_{t,j} \xrightarrow{\text{I.}} x_{mig} \xrightarrow{\text{II.}} x_{mig,sel} \xrightarrow{\text{III.}} x_{mig,sel,drift} = x_{t+1,j}$$

The variable $x_{mig,sel}$ represents the expected frequency of the allele in generation *t+1*. Drift is the random deviation of the actual frequency of the allele from this expectation. To determine the actual frequency of the allele in the next generation, $x_{t+1,j}$, I generate random numbers from the Inverse Binomial distribution with mean $N_e x_{mig,sel}$ and variance $N_e x_{mig,sel}(1-x_{mig,sel})$ using the following procedure:

I. Random number generator Philox (Salmon *et al.* 2011) generates a uniform random number between 0 and 1.
II. If $N_e x_{mig,sel} \leq 6$, then that uniform variable is fed into the exact Inverse Poisson CDF as an approximation to the Binomial.
III. If $N_e x_{mig,sel} > 6$, then a Normal approximation to the Binomial is used.

As $N_e = 2N/(1+F)$, inbreeding affects drift as well as selection.

Adding New Mutations

Using the glossary from Table 1, for population *j* in generation *t*:

$$\mu = \mu(j,t), \quad N_e = 2\mathrm{N}(j,t)/(1+F)$$

$$14)\ \lambda_\mu = N_e \mu L$$

$$\text{starting frequency, } x = 1/N_e$$

The Poisson Random Field shares an important assumption with Watterson's infinite sites model in that regardless of how many sites are currently polymorphic, mutations will never strike a currently polymorphic site and the number of monomorphic sites that a mutation can occur at is always the total number of sites, *L* (Watterson 1975, Sawyer and Hartl 1992). Eq. 14 defines the expected number of mutations in population *j* for generation *t+1*. The actual number of new mutations is drawn from the Inverse Poisson distribution using the same procedure detailed in *Simulation Initialization*. New mutations can be added to generation *t+1* in parallel and simultaneously with the new frequency

calculations. Each new mutation is given a 4-part unique ID consisting of the thread and compute device that birthed it (if more than one graphics card is used) as well as the generation and population in which it first arose.

Compact

The general compact algorithm is outlined in Figure 2C). GO Fish's version uses a more advanced variant to speed up compaction and lower its memory requirement as discussed in (Billeter *et al.* 2009) and adapted from (Bakunas-Milanowski *et al.* 2015).

References

Bakunas-Milanowski D, Rego V, Sang J, Yu C. (2015) A fast parallel selection algorithm on GPUs. IEEE 2015 International Conference on Computational Science and Computational Intelligence (CSCI): 609-614.

Billeter M, Olsson O, Assarsson U. (2009) Efficient stream compaction on wide SIMD many-core architectures. ACM Proceedings of the conference on high performance graphics 2009: 159-166.

Hardy GH. (1908) Mendelian proportions in a mixed population. Science 28(706): 49-50.

Higham NJ. (1993) The accuracy of floating point summation. SIAM Journal on Scientific Computing 14(4): 783-799.

Kimura M. (1964) Diffusion models in population genetics. J. Appl. Prob. 1(2): 177-232.

Merrill D. (2016) CUB. v. 1.6.4 [ONLINE] Available at: https://nvlabs.github.io/cub/. Accessed on Aug. 12, 2017.

Nagylaki T. (1980) The strong-migration limit in geographically structured populations. J. Math. Biol. 9(2): 101-114.

Salmon JK, Moraes M, Dror RO, Shaw DE. (2011) Parallel random numbers: As easy as 1, 2, 3. IEEE High Performance Computing, Networking, Storage and Analysis (SC), 2011 International Conference for: 1-12.

Sawyer SA, Hartl DL. (1992) Population genetics of polymorphism and divergence. Genetics 132(4): 1161-1176.

Watterson G. (1975) On the number of segregating sites in genetical models without recombination. Theor. Popul. Biol. 7(2): 256-276.

Weinberg W. (1908) Über den nachweis der vererbung beim menschen. Jahresh Wuertt Ver Vaterl Natkd 64: 369-382.
