# Supplementary material for: Accelerating Wright–Fisher Forward Simulations on the Graphics Processing Unit
Source: G3 (Bethesda). 2017 Aug 2;7(9):3229–36. doi: 10.1534/g3.117.300103 (PMC5592947; doi:10.1534/g3.117.300103)
Supplement: Supplementary file 2 [file 3229FigureS1.pdf]

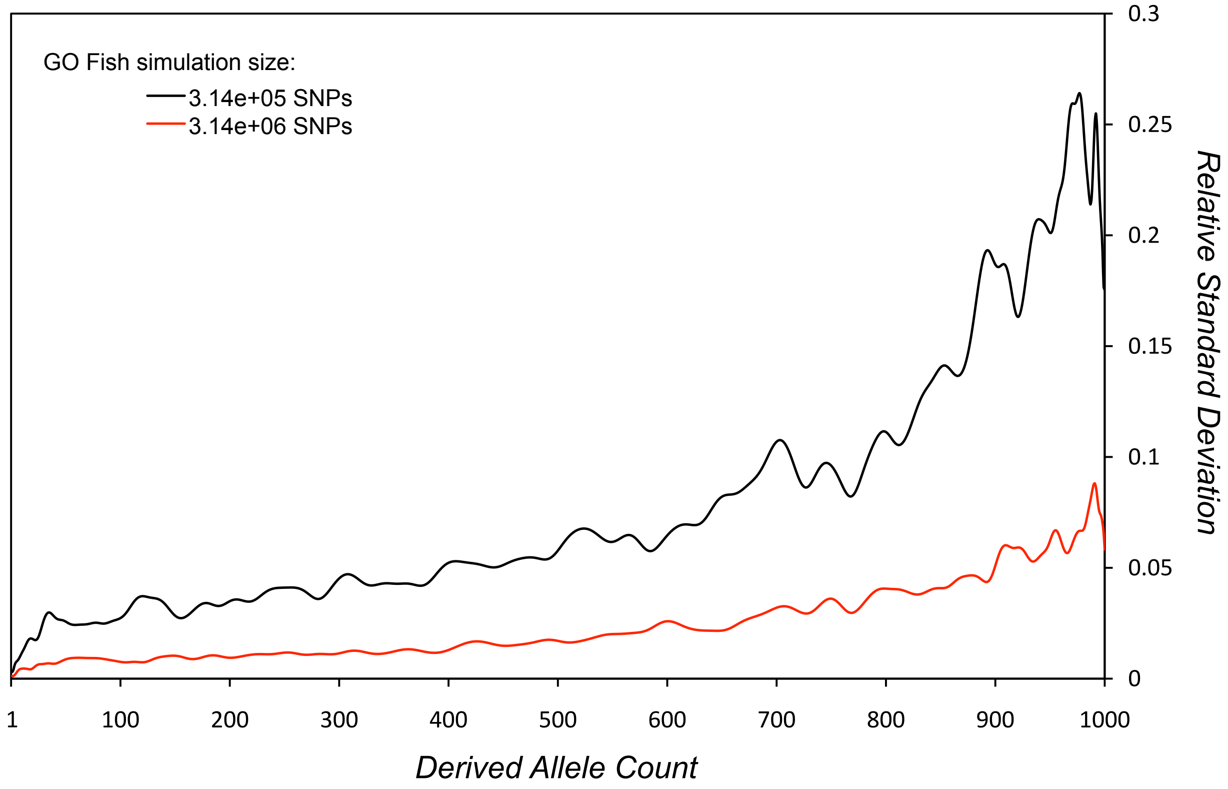

Figure S1. Dispersion of simulation runs over the SFS. Figure 5 shows the average SFS of 50 GO Fish simulation runs with ~3x10^6 SNPs for a complex, multi-population evolutionary scenario – i.e. the mean estimated probability of seeing a SNP at each frequency in the sample of 1001 EU genomes given the evolutionary history. Dispersion of the results of these simulation runs for each allele count is measured by the relative standard deviation (σ/μ) and is displayed on the red line. The black line is the same evolutionary & sampling scenario but with a smaller simulation size, only ~300,000 SNPs. As the mean probability of seeing a SNP at a frequency in the sample becomes more rare, the variance in the estimate of that probability across simulation runs increases. For example: at a derived allele count (DAC) of 1, the dispersion is ~0.001/0.003 (red/black), whereas at a DAC of 1000, the dispersion is ~0.058/0.175 (red/black). The factor of 10 separating the two simulation sizes engenders a roughly 3-fold difference in the dispersion (σ being the square root of the variance). Thus the expected precision with which an individual simulation run of 3x10^7 SNPs can ascertain the probability of seeing a SNP at a DAC of 1000 would be just under 2% for this evolutionary & sampling scenario. How much noise in the results of a simulation is acceptable for a particular application will determine the necessary simulation size.
